# National survey of the rat hepatitis E virus in rodents in Spain, 2022 to 2023

**DOI:** 10.2807/1560-7917.ES.2025.30.12.2400473

**Published:** 2025-03-27

**Authors:** Javier Caballero-Gómez, Tomás Fajardo-Alonso, Lucía Ríos-Muñoz, Adrián Beato-Benítez, María Casares-Jiménez, Ignacio García-Bocanegra, Raúl Cuadrado-Matías, Alba Martí-Marco, Javier Martínez, Remigio Martínez, Eva Martínez Nevado, Francisco Ruiz-Fons, João Rodrigo Mesquita, Màrius Fuentes, Diana Corona-Mata, Moisés Gonzálvez, Víctor Lizana, Pilar Soriano, Pilar Foronda, Dietmar Crailsheim, Antonio Rivero-Juárez, Antonio Rivero

**Affiliations:** 1 Grupo de Virología Clínica y Zoonosis, Unidad de Enfermedades Infecciosas, Instituto Maimónides de Investigación Biomédica de Córdoba (IMIBIC), Hospital Universitario Reina Sofía, Universidad de Córdoba, Córdoba, Spain; 2 CIBERINFEC, ISCIII—CIBER de Enfermedades Infecciosas, Instituto de Salud Carlos III, Madrid, Spain; 3 Departamento de Sanidad Animal, Grupo de Investigación en Sanidad Animal y Zoonosis (GISAZ), UIC Zoonosis y Enfermedades Emergentes ENZOEM, Facultad de Veterinaria, Universidad de Córdoba, Córdoba, Spain; 4 Health & Biotechnology (SaBio) group, Instituto de Investigación en Recursos Cinegéticos IREC (CSIC—UCLM—JCCM), Ciudad Real, Spain; 5 Servicio de Análisis, Investigación y Gestión de Animales Silvestres (SAIGAS), Veterinary Faculty, Universidad CEU-Cardenal Herrera, Alfara del Patriarca, Valencia, Spain; 6 Bioparc Valencia, Valencia, Spain; 7 Veterinary Department, Madrid-Zoo Aquarium, Madrid, Spain; 8 ICBAS – School of Medicine and Biomedical Sciences, Porto University, Porto, Portugal; 9 Epidemiology Research Unit (EPIUnit), Instituto de Saúde Pública da Universidade do Porto, Porto, Portugal; 10 Laboratório para a Investigação Integrativa e Translacional em Saúde Populacional (ITR), Porto, Portugal; 11 Parasites & Health Research Group, Department of Pharmacy, Pharmaceutical Technology and Parasitology, Faculty of Pharmacy and Food Sciences, University of Valencia, Burjassot, Valencia, Spain; 12 Departamento de Sanidad Animal, Facultad de Veterinaria, Campus de Excelencia Internacional Regional “Campus Mare Nostrum”, Universidad de Murcia, Murcia, Spain; 13 Rio Safari Elche, Alicante, Spain; 14 Department Obstetricia y Ginecología, Pediatría, Medicina Preventiva y Salud Pública, Toxicología, Medicina Legal y Forense y Parasitología, Universidad de La Laguna, Tenerife, Canary Islands, Spain; 15 Instituto Universitario de Enfermedades Tropicales y Salud Pública de Canarias (IUETSPC), Universidad de La Laguna, Tenerife, Canary Islands, Spain; 16 Research Department, Fundació Mona, Girona, Spain

**Keywords:** rocahepevirus, rat hepatitis E virus, rats, public health, zoonoses, emerging

## Abstract

**Background:**

Rat hepatitis E virus (ratHEV) is an emerging virus causing acute and chronic hepatitis in humans. Rats are the main natural reservoir of this pathogen. Large-scale studies assessing ratHEV circulation in rodents in Spain are lacking.

**Aim:**

We aimed to determine the prevalence of ratHEV in rats in Spain and evaluate potential transmission risk to humans.

**Methods:**

We designed a cross-sectional nationwide study where black (*Rattus rattus*) and Norway (*R. norvegicus*) rats were collected and analysed between 2022 and 2023 for ratHEV infection using real-time (RT)-qPCR testing of liver tissue. Sequencing and analysis of ratHEV shedding in faeces were carried out in positive animals.

**Results:**

RatHEV was detected in 125 of the 481 rats analysed, supposing a prevalence of 26.0% (95 CI%: 22.3–30.1). Positive rats were found in urban (25.6%), and farm (29.8%) settings. Black rats (31.3%) had 1.5 times higher odds of being infected by the virus than Norway rats (22.5%) (p = 0.049). Significantly higher prevalence of ratHEV was detected in rodents sampled from southern (31.9%) than northern (17.8%) Spain (p = 0.003). Viral RNA was detected in faeces from 45.5% of infected rats. Phylogenetic analysis evidenced a wide genetic diversity of ratHEV sequences, some showing high homology with ratHEV strains found in patients from Spain.

**Conclusions:**

Circulation of ratHEV appears to be heterogeneous and the virus appears to be endemic among rat populations in Spain, highlighting the possible risk of zoonotic transmission of this emerging virus in this country.

**Key public health message**

**What did you want to address in this study and why?**

Since the first human cases of rat hepatitis E virus (ratHEV) in Europe were detected in Spain in 2022, additional cases have been reported in this country. We wanted to determine how common the virus was in rats, that are the main carriers, and to assess transmission risks.

**What have we learnt from this study?**

We found that ratHEV is common and occurs throughout Spain in rats, even in those that live close to people. The virus found in rats is very similar to the one found in European patients with viral hepatitis. We also learned that the virus is not evenly spread in the country; there appear to be more infected rats in some areas than in others and nearly half of the infected rats shed the virus in their faeces.

**What are the implications of your findings for public health?**

This study demonstrates that ratHEV might be an infectious disease risk for humans in Spain. There appears to be a need to implement effective diagnostic and surveillance protocols to obtain a better picture of the epidemiology of the disease in humans. Proper control measures, including rodent control and waste management, should be implemented to minimise the risk of human exposure to ratHEV.

## Introduction

Rat hepatitis E virus (ratHEV) (family *Hepeviridae*), also known as *Rocahepevirus ratti*, is an emerging and globally distributed virus that can cause acute and chronic hepatitis in humans, sometimes leading to fatal outcomes [1-3]. Rodents, particularly black (*Rattus rattus*) and Norway (*Rattus norvegicus*) rats, are the main reservoirs of ratHEV, and direct or indirect contact with these species has been suggested as a potential transmission route [4,5], supported by the high homology found between ratHEV strains from rats and humans [6]. Since the first European ratHEV cases were detected in Spain in 2022 [3], the number of humans infected or exposed to this virus has increased in Europe over the last couple of years, with 12 cases (three infected and nine exposed) reported in 2023 and 17 (two infected and 17 exposed) reported in 2024 [7-11]. Despite this, large-scale studies assessing ratHEV circulation in rodents in Spain, which could facilitate the molecular traceability of human cases and improve our understanding of the epidemiology of ratHEV in its main reservoir, are lacking. In this respect, molecular epidemiological studies allow the assessment of the genetic diversity of ratHEV and the identification of genetic variants, enabling the tracing of potential routes of transmission and/or the identification of potential hotspots. We therefore aimed to determine the prevalence of ratHEV in rats in Spain and evaluate potential transmission risks to humans.

## Methods

### Study design and sampling

We designed and carried out a cross-sectional nationwide study in which rats (*R. rattus* and *R. norvegicus*) were sampled between 2022 and 2023 with the aim of assessing the prevalence of infection with ratHEV. The sample size was calculated assuming a ratHEV prevalence of 50%, which provides the highest sample size in studies where prevalence is unknown, a 95% confidence level (CL) and a desired precision of ± 5% [12]. Thus, a minimum sample size of 385 rodents was obtained. The outcome variable (criterion for a sample to be considered positive) was the detection of ratHEV RNA in liver tissue by either one of two qPCR methods [2,13]. Geographically, Spain was latitudinally divided into three areas (north, centre and south) on the basis of climatic characteristics (details of the division are included in the Supplementary Material), and at least 99 rats were sampled in each area to detect infection with a 95% CL, assuming a minimum within-area prevalence of 3%. Sampling was carried out in collaboration with ongoing rodent control campaigns conducted by different pest control companies in urban and farm settings throughout the country. Additional information regarding sampling can be found in the Supplementary Material).

Carcasses of rats were frozen at -20 °C and shipped to the Department of Animal Health at the University of Cordoba (ROR code 05yc77b46). Liver tissue and, whenever possible, faecal samples from the rectum were collected from all rat carcasses and stored at -80 °C until analysis. The epidemiological information of each sampled individual, such as its species (based on external morphology), age, sex, habitat, sampling date and sampling location, was recorded (Table 1). Rat age was estimated based on tooth eruption, reproductive status and body size [14].

**Table 1:** Epidemiological information and characteristics of the rat population studied, Spain, 2022–2023 (n = 481)

Variable	Category	n	%
Species	Norway rat	289	60.1
	Black rat	192	39.9
Age^a^	Adult	390	81.1
	Young	91	18.9
Sex	Female	257	53.4
	Male	224	46.6
Habitat	Urban environment	434	90.2
	Farm environment	47	9.8
Sampling year	2022	311	64.7
	2023	170	35.3
Sampling area^b^	North	146	30.4
	Centre	131	27.2
	South	204	42.4

^a^ Rat age was estimated based on tooth eruption, reproductive status and body size [14].

^b^ Spain was latitudinally divided into three areas (north, centre and south) on the basis of climatic characteristics.

### Molecular analysis

RNA from liver tissue was extracted via the RNeasy Mini Kit (QIAGEN, Hilden, Germany) using an automated procedure (QIAcube, QIAGEN), and screening for ratHEV infection was conducted in parallel using two previously described real-time (RT)-qPCR methods that target open reading frame (ORF)1 of ratHEV [2,13]. These screenings were conducted using the Takara qPCR master mix in a CFX Connect instrument (Bio-Rad, Hercules, United States (US)). Positive samples were then tested via nested RT‒PCRs in which two segments of ratHEV, located at the beginning and end of ORF1, were amplified. Samples positive for these nested RT‒PCRs were sequenced. All primers and probe sets employed in the study, as well as additional data about the molecular assays, can be found in Supplementary Table S1.

Additionally, in rats with detectable ratHEV RNA in liver tissue, whenever available, the presence of the virus was evaluated in faecal samples to assess the potential excretion of ratHEV. Therefore, viral RNA was evaluated following the same qPCR protocol as described above, but the RNA was extracted using the IndiSpin Pathogen Kit (Indical Bioscience, Leipzig, Germany).

### Statistical analysis

The prevalence of ratHEV in our sample was calculated by dividing the number of cases by the total number of animals tested via two-sided exact binomial 95% confidence intervals (CI). Associations between the presence of ratHEV and the explanatory variables were first analysed via Fisher's exact test or Pearson's chi-squared test, as appropriate. Variables with p values lower than 0.05 were included in multivariate analyses. A regression model was used to assess the effect of the variables selected in the bivariate analysis. Statistical analyses were performed via SPSS version 25.0 (IBM SPSS Inc., Chicago, US).

Confirmation of the presence of RatHEV was performed using the HEVnet typing tool (https://www.rivm.nl/mpf/typingtool/hev/) and confirmed via BLAST analysis. A phylogenetic tree was constructed via the maximum likelihood method using MEGA X software [15] via the bootstrap method (with 1,000 replicates).

## Results

### Study population

A total of 481 rats were collected nationwide, including 146 from northern Spain, 131 from central Spain and 204 from southern Spain. Detailed information about the sampled population is shown in Table 1. Among the rats, 390 were adults (81.1%) and 257 were females (53.4%). In terms of species, a total of 289 (60.1%) specimens were identified as Norway rats and 192 (39.9%) were black rats. The study population included rodents from urban (90.2%) and farm (9.8%) settings.

### Prevalence of rat hepatitis E virus

We detected ratHEV in the liver tissue of 125 of the 481 animals analysed, with a prevalence of 26.0% (95% CI: 22.3–30.1) (Table 2). By species, the prevalence was 22.5% (95% CI: 18.1–27.7) in Norway rats and 31.3% (95% CI: 25.1–38.1) in black rats. We found positive animals in all three habitat settings sampled: urban (25.6%), and farm (29.8%), as well as in all three sampling regions. The regression model identified the variables ‘species’ and ‘sampling region’ as hazard factors potentially associated with ratHEV infection in rats in Spain (Table 2). Compared with Norway rats, black rats were 1.5 times (odds ratio (OR)) more likely to be infected by the virus (p = 0.049; 95% CI: 1.0–2.3). In addition, we detected a significantly greater prevalence in rodents sampled from southern Spain (31.9%) than in those from northern Spain (17.8%) (p = 0.003; OR = 2.2; 95% CI: 1.3–3.7).

**Table 2:** Prevalence of rat hepatitis E virus in rats according to explanatory variables and the results of statistical analyses, Spain, 2022–2023

Variable	Categories	Overall prevalence in % (95% CI)	No positives/no analysed	p value of bivariate analyses	OR (95% CI)	p value of multivariate analyses
Species	Black rat	31.3 (25.1–38.1)	60/192	**0.021**	1.5 (1.0–2.3)	**0.049**
	Norway rat	22.5 (18.1–27.7)	65/289		Reference	
Age	Adult	26.7 (22.5–31.3)	104/390	0.287	NA	NA
	Young	23.1 (15.6–32.7)	21/91			
Sex	Female	28.8 (23.2–34.2)	73/257	0.117	1.3 (0.8–1.9)	0.298
	Male	23.2 (18.2–29.2)	52/224		Reference	
Habitat	Urban environment	25.6 (21.7–29.9)	111/434	0.320	NA	NA
	Farm environment	29.8 (18.7–44.0)	14/47			
Sampling year	2022	25.1 (20.6–30.2)	78/311	0.306	NA	NA
	2023	27.6 (21.5–34.8)	47/170			
Sampling region ^a^	North	17.8 (12.5–24.8)	26/146	**0.013**	Reference	NA
	Centre	26.0 (19.2–34.1)	34/131		1.7 (0.9–3.0)	**0.085**
	South	31.9 (25.9–38.5)	65/204		2.2 (1.3–3.7)	**0.003**

CI: confidence interval; NA: not applicable; OR: odds ratio.

^a^ Spain was latitudinally divided into three areas (north, centre and south) on the basis of climatic characteristics.

Significant p values are displayed in bold.

Faeces were available for 112 (89.6%) of the 125 infected animals. Viral RNA was detected in 51 rats, with a frequency of detection of 45.5% (51/112). We detected positivity in the faeces of 50.9% (28/55) and 40.4% (23/57) of the Norway and black rats, respectively, sampled and in both adult (45.7%; 43/94) and young (42.0%; 8/19) individuals. Geographically, animals shedding ratHEV were collected across all three regions of Spain, with frequencies of 33.3% (8/24), 62.5% (20/32) and 41.1% (23/56) in the northern, central and southern regions, respectively.

### Phylogenetic analyses

**Figure 1:**
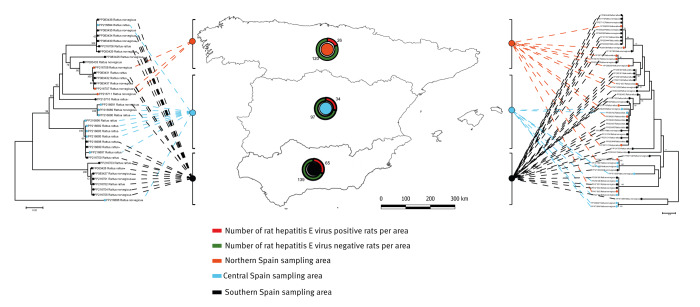
Spatial distribution of rat hepatitis E virus sequences and frequency of positivity by sampling area, Spain, 2022–2023

**Figure 2:**
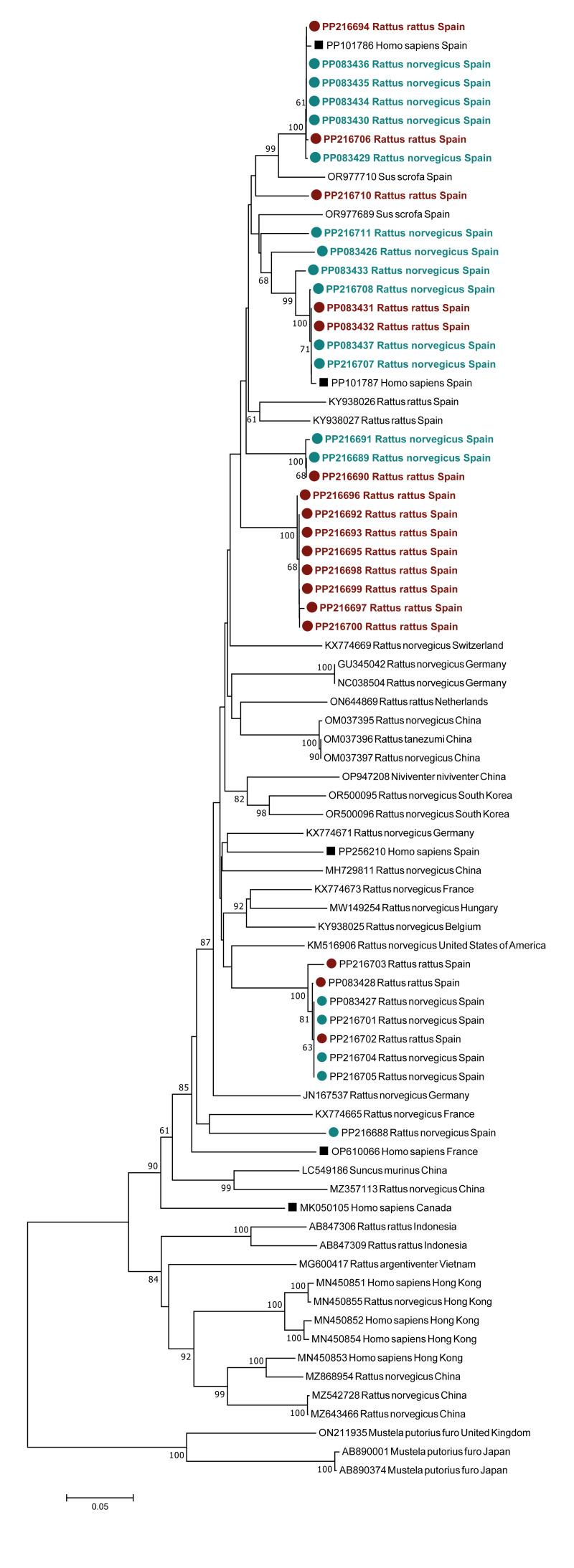
Phylogenetic tree constructed via the neighbour-joining method (1,000 replicates)

We obtained viral sequences from 105 (80.0%) of the 125 ratHEV positive rats. The GenBank accession numbers of all the sequences are presented in Supplementary Table S2. Phylogenetic analyses revealed high genetic diversity of the ratHEV sequences, with *p* distances ranging from 0 to 0.221. Two phylogenetic trees were constructed using the sequences obtained in the present study to assess potential spatial clustering. Each of them was constructed with the partial genomes obtained at the beginning or end of ORF1 of ratHEV (Figure 1). No associations were detected between the ratHEV sequences obtained in the present study and the respective sampling areas. In addition, we compared the sequences from positive rodents with those previously retrieved from infected animals and patients from Spain and other countries publicly available in the GenBank database (Figure 2). The BLAST analyses revealed nucleotide identities ranging from 88% to 99% and from 85% to 88% with other ratHEV sequences from rodents, pigs and humans from Spain and other European countries, respectively. The phylogenetic tree constructed revealed that our sequences clustered in different groups next to other phylogenetically related ratHEV strains obtained from animals and humans from Spain, France, Germany, The Netherlands, China, Switzerland and the US (Figure 2).

Neighbour-joining phylogenetic trees were inferred from alignment of the sequences obtained in the present study via nested RT‒PCR-1 (left) and nested RT‒PCR-2 or 3 (right).

The sequences obtained in the present study are in bold (blue: Norway rat (*Rattus norvegicus*); red: black rat (*Rattus rattus*)). The rat hepatitis E virus (ratHEV) sequences obtained from humans are indicated with black squares.

The percentage of replicate trees in which the associated taxa clustered together in the bootstrap test (1,000 replicates) is shown next to the branches when values were greater than 60. The tree is drawn to scale, with branch lengths in the same units as those of the evolutionary distances used to infer the phylogenetic tree. The analysis involved 78 nt sequences and 711 positions in the final dataset (nt 4176–4886 using GU345042 as a reference strain of ratHEV).

## Discussion

Rodents are widely recognised as important synanthropic reservoirs of zoonotic viruses that have threatened human health throughout history [16,17]. In 2010, a new hepevirus closely related to hepatitis E virus was discovered in rodents from Germany [18], and it currently represents an emerging virus of increasing public health interest [1]. During the last two decades, studies in different European countries revealed prevalences of ratHEV ranging between 2.6% and 20.6% in rats [19-24]. Here, we provide evidence that ratHEV is frequent in synanthropic rats from Spain, representing a potential zoonotic risk. This is further supported by the high homology detected between the ratHEV sequences obtained in the present study and those retrieved from patients with acute or chronic hepatitis in Europe.

The zoonotic route of the virus is not yet fully understood. Our results confirm that the virus is shed in faeces by nearly half of infected rats. These findings reinforce our hypothesis that exposure to rodent droppings could be a potential source of ratHEV infection in humans and other animal species, which in turn may potentially act as intermediate hosts or turn into secondary reservoirs of ratHEV. In this respect, the recent detection of ratHEV in faeces from farmed pigs might be associated with ratHEV infection in sympatric rats as supported by the high prevalence detected in pest rats from farms in our study [25]. Overall, these findings, together with the wide ratHEV circulation in rats throughout Spain, echo the serological and molecular results previously reported in humans [3,7-9], since human exposure to ratHEV has been detected in northern, central and southern Spain. These findings call for further study and implementing effective diagnostic and surveillance protocols.

In contrast to the geographical pattern of ratHEV observed by Palombieri et al. [26], a high diversity of ratHEV strains was detected in rodent populations throughout Spain. They were distributed in different clades along the constructed phylogenetic trees, and most of the sequences clustered independently of whether they originated from Norway or black rats, supporting the previous hypothesis of ratHEV transmission among these species [19]. Moreover, this genetic diversity was also found within the sampling regions, with strains being found in up to seven different clades in the same study area. This high diversity might also explain the varying sensitivity of each RT-qPCR assay for detecting ratHEV. Our results confirm the wide genetic evolution of this virus, even within each area, and highlight the need to monitor ratHEV within rat populations in Spain and other countries [6]. Given that ratHEV is shed mainly in the faeces of rodents [27], surveillance in wastewater has been suggested as an alternative noninvasive option to monitor ratHEV [7,26,28], although the usefulness of this approach requires further study.

We observed similar frequencies of positivity in the two habitats analysed, suggesting that densities of humans or livestock, which are usually high in urban areas and farms, may not have an effect on ratHEV occurrence as previously suggested [19]. To date, very few studies have successfully identified potential hazard factors associated with ratHEV infection in rodents [29-31]. Interestingly, black rats had higher odds of being infected by ratHEV than Norway rats in the present study. This may be due to two possible explanations: (i) ecological or behavioural variations between the two species (e.g. habitat or feeding behaviours) [32], which may lead to different exposure levels, or (ii) differing susceptibility to ratHEV strains found in Spain. On the other hand, a significantly greater prevalence of ratHEV was detected in rodents from southern Spain than in those from northern Spain, indicating possible differences in the circulation of the virus in rodents across the country. Consequently, the transmission risk of ratHEV to other sympatric species, including humans, may potentially be greater in southern Spain than in northern Spain. Although the transmission dynamics of ratHEV remain poorly understood, limited environmental hygiene or pest control measures have been associated with an increased risk of ratHEV circulation in rats, probably due to the increased abundance of rats in these regions [29,33].

Several limitations should be noted. In the present study, we used two qPCR assays that had previously been validated and demonstrated to be sensitive tools for ratHEV screening [[34]]. Indeed, we detected a high prevalence of ratHEV in our cohort of rats using this combined screening approach. However, the detection limits of both assays were not evaluated, and the potential presence of infected rats that may have escaped detection cannot be ruled out. Additionally, sampling in this study was not spatiotemporally homogeneous. These limitations underscore the need for caution when interpreting our findings and highlight areas for future research to address these gaps.

## Conclusion

In this cross-sectional study of rodents in Spain, we provide further evidence that ratHEV appears to be endemic in the country and that there may be a risk of zoonotic transmission of this emerging virus. Our results also point towards the heterogeneous circulation of ratHEV within rat populations across Spain and suggest that risk of zoonotic transmission of this virus might be greater in southern Spain than in northern Spain. Control measures and protocols, including effective rodent control programs, adequate control of both waste items and availability of animal or human food sources, should be implemented to minimise the risk of exposure to ratHEV for humans and other animals. Furthermore, we suggest large-scale surveillance programs of ratHEV in rodents in Spain, as well as in other European countries, to further assess and better understand the risk to public health.

## Supplementary Data

401000 Supplementary Material
