# Supplementary material for: National survey of the rat hepatitis E virus in rodents in Spain, 2022 to 2023
Source: Euro Surveill. 2025 Mar 27;30(12):2400473. doi: 10.2807/1560-7917.ES.2025.30.12.2400473 (PMC11951417; doi:10.2807/1560-7917.ES.2025.30.12.2400473)
Supplement: Supplementary Material [file 24-00473_RIVERO-JUAREZ_Supplement.pdf]

Supplementary material

This supplementary material is hosted by *Eurosurveillance* as supporting information alongside the article “National survey of the rat hepatitis E virus in rodents in Spain, 2022 to 2023”, on behalf of the authors, who remain responsible for the accuracy and appropriateness of the content. The same standards for ethics, copyright, attributions and permissions as for the article apply. Supplements are not edited by *Eurosurveillance* and the journal is not responsible for the maintenance of any links or email addresses provided therein

Additional data about molecular assays

Screening of ratHEV samples were carried out using real-time RT-qPCR with the primers and probes described previously[1,2] and listed in Table S1. They were performed in final volumes of 50µl containing 20µl of the One Step PrimeScript III RT-qPCR Mix (Takara Bio, Shiga, Japan), 17.75µl of RNase-DNase free water, 0.75µl of forward (20µM), 0.75µl of reverse (20µM), 0.75µl of probe (20µM) and 10µl of RNA. As positive control, we used RNA from a rodent liver sample previously tested as ratHEV positive in our laboratory (GenBank accession number: OR282813). Positive liver samples by RT-qPCR were then sequenced by nested RT-PCR using the primers depicted in Table S1. Samples were tested serially from nested RT-PCR-1 to 4 until positive results were obtained. The first round of nested RT-PCR was conducted using the One Step PrimeScript III RT-PCR Kit (Takara Bio, Shiga, Japan) using the same positive control than for RT-qPCR, as well as volumes for primers, master mix and RNA as described but adding 18.5 µl of RNase-DNase free water. The second round was carried out with the premixed 2× solution containing Taq DNA Polymerase, dNTPs and Reaction Buffer (Promega, Madison, WI, USA). The volumes for each reaction were: 25µl of master mix, 15µl of RNase-DNase free water, 2.5µl

of each primer (20µM each) and 5µl of template., whereas the second was carried out with the premixed 2X solution of Taq DNA Polymerase, dNTPs and Reaction Buffer (Promega).

Table S1. List of primers and probes, when applicable, for each molecular assay

PCR	ID	Ref	Primers (5'-3')		Probe (5'-3')
			**Forward**	**Reverse**	
RT-qPCR	qPCR-1	1	CTTGTTGAGCTYTTCTCCCCT	CTGTACCGGATGCGACCAA	TGCAGCTTGTCTTTGARCCC
RT-qPCR	qPCR-2	2	CCACGGGGGTTAATACTGC	CGGATGCGACCAAGAAACAG	CGGCTACCGCCTTTGCTAATGC
Nested RT-PCR	seqPCR-1	3	1st PCR:<br>CTTGGTTYAGGGCCATAGAG<br>2nd PCR:<br>TTYAGGGCCATAGAGAAGGC	1st PCR:<br>CAGCAGCGGCACGAACAGCA<br>2nd PCR:<br>ACAGCAAAAGCACGAGCACG	-
Nested RT-PCR	seqPCR-2	-	1st PCR:<br>TTTGCTAATGCTCAGGTGGT<br>2nd PCR:<br>CCTYTGCAGCTTGTCTTTGA	1st PCR:<br>CATDCCATGAGCACGCAT<br>2nd PCR:<br>GTGCAAAAGGAAAGATCAG	-
Nested RT-PCR	seqPCR-3	4	1st PCR:<br>CCTYTGCAGCTTGTCTTTGA<br>2nd PCR:<br>CTGTTTCTTGGTCGCATCCG	1st PCR:<br>CATDCCATGAGCACGCAT<br>2nd PCR:<br>GTGCAAAAGGAAAGATCAG	-

Additional data about results

Table S2. Molecular results of positive animals for rat hepatitis E virus.

ID	qPCR-1*	Ct qPCR-1	qPCR-2*	Ct qPCR-2	GenBank Accesion Number	Faeces global qPCR	qPCR-1	Ct qPCR-1	qPCR-2	Ct qPCR-2
1	+	20.50	+	20.29	PP216692	+	+	22.48	+	25.37
2	-		+	39.17		-	-		-	
3	-		+	39.87	PP507100	-	-		-	
4	-		+	41.08	PP507101	-	-		-	
5	-		+	36.96	PP256190	-	-		-	
6	+	16.43	+	19.02	PP216688	+	+	23.28	+	26.08
7	+	33.24	-		PP256186	+	+	33.31	+	37.44
8	+	30.73	+	35.46	PP507098	-	-		-	
9	+	31.45	+	35.16	PP256187	+	+	39.92	-	
10	+	33.06	+	37.28		-	-		-	
11	+	30.81	+	36.50	PP256188	+	+	42.92	-	
12	-		+	38.03	PP256189	-	-		-	
13	+	11.05	+	15.61	PP216689	+	+	21.32	+	25.36
14	+	26.99	+	32.07	PP216690	+	+	34.09	-	
15	+	24.47	+	29.48	PP216691	+			+	38.13
16	+	31.44	+	37.10	PP507099	-	-		-	
17	+	34.70	+	38.64		-	-		-	
18	+	32.57	+	32.95	PP216698	-	-		-	
19	+	17.21	+	18.11	PP216693	+	+	19.48	+	22.61
20	-		+	37.40	PP256193	+	+	34.84	+	41.07
21	-		+	34.53	PP216699	+	+	35.92	+	39.27
22	+	33.79	+	33.69	PP216700	-	-		-	
23	-		+	37.06	PP256191	+	+	34.34	+	36.95
24	-		+	38.16	PP216694	-	-		-	
25	+	30.09	+	30.10	PP216695	+	+	29.80	+	32.10
26	+	24.82	+	25.04	PP216696	+	+	25.97	+	31.09
27	-		+	39.92	PP256192	+	-		+	38.10
28	+	32.27	+	32.95	PP216697	+	+	32.61	+	35.55
29	+	**22.75**	-		PP471864					

ID	qPCR-1*	Ct qPCR-1	qPCR-2*	Ct qPCR-2	GenBank Accesion Number	Faeces global qPCR	qPCR-1	Ct qPCR-1	qPCR-2	Ct qPCR-2
30	-		+	39.37		+	+	44.65		
31	-		+	39.76	PP507102	-	-		-	
32	+	36.3	-		PP256196	+	+	35.63	-	
33	-		+	40.54		+	+	36.03	-	
34	+	36.4	+	38.75		-	-		-	
35	-		+	39.40	PP471853	-	-		-	
36	-		+	38.18		+	+	36.60	-	
37	-		+	39.54						
38	+	36.51	+	38.91	PP256207	-	-		-	
39	+	26.79	-		PP471854	+	+	29.61	-	
40	+	33.02	-		PP083440	+	+	36.56	+	38.49
41	+	27.96	+	31.13		+	+	33.73	+	36.67
42	+	27.29	+	32.56	PP083425	+	+	28.80	+	33.76
43	+	13.30	+	19.90	PP083427	+	+	18.86	+	21.72
44	+	28.69	+	32.60	PP216701	+	+	32.72	-	
45	+	33.86	+	36.07	PP216702	-	-		-	
46	+	36.41	-			-	-		-	
47	+	28.45	-			-	-		-	
48	+	28.07	+	34.86	PP216703	+	+	33.19	-	
49	+	25.26	+	33.51	PP216704	+	+	35.48	-	
50	+	25.27	-		PP216705	-	-		-	
51	+	27.38	+	32.01	PP216706	+	+	36.94	-	
52	+	29.10	+	34.27	PP083428	+	+	34.25	+	36.61
53	+	28.83	+	36.05	PP083441	+	+	35.99	-	
54	+	27.20	+	31.54	PP083429	+	+	32.45	+	41.10
55	+	28.27	+	32.86	PP083430					
56	+	27.18	+	35.50	PP083431	+	+	35.88	-	
57	+	14.74	+	33.53	PP083442					
58	+	23.40	+	20.95	PP083432					
59	+	13.26	+	29.41	PP083433	-	-		-	

ID	qPCR-1*	Ct qPCR-1	qPCR-2*	Ct qPCR-2	GenBank Accesion Number	Faeces global qPCR	qPCR-1	Ct qPCR-1	qPCR-2	Ct qPCR-2
60	+	**13.27**	+	**19.08**	**PP083434**					
61	+	**30.51**	+	**19.07**	**PP083435**	+	+	21.09	+	23.33
62	+	**28.60**	+	**37.82**	**PP083436**					
63	+	**28.27**	+	**34.66**	**PP083443**	-	-		-	
64	+	**29.86**	+	**34.10**	**PP083444**	+	+	38.42	-	
65	+	**30.02**	+	**37.21**	**PP101769**	-	-		-	
66	+	**31.32**	+	**36.46**	**PP083445**	-	-		-	
67	+	**32.52**	+	**38.33**	**PP083446**	+	+	35.81	-	
68	+	**30.25**	-		**PP083447**	-	-		-	
69	+	**29.41**	+	**36.73**	**PP083448**	-	-		-	
70	+	**32.92**	+	**38.03**	**PP083449**	-	-		-	
71	+	**30.65**	+	**39.27**	**PP101770**	-	-		-	
72	-		+	**37.69**	**PP083450**	-	-		-	
73	+	**35.47**	-		**PP083451**	-	-		-	
74	-		+	**39.27**	**PP083452**	-	-		-	
75	+	**34.66**	-		**PP083453**	-	-		-	
76	+	**25.31**	+	**30.76**	**PP083437**	+	+	40.52	+	41.44
77	+	**34.53**	+	**43.74**	**PP101760**					
78	+	**34.23**	+	**36.74**	**PP216707**	+	+	36.49	-	
79	+	**31.44**	+	**36.57**	**PP256197**	+	+	34.54	+	41.58
80	-		+	**40.27**		-	-		-	
81	+	**33.44**	+	**39.51**	**PP256198**	+	-		+	38.86
82	+	**35.71**	-		**PP996732**	+	+	36.07	-	
83	+	**35.01**	-		**PP256199**	+	+	34.35	-	
84	+	**32.25**	-		**PP256200**	-	-		-	
85	+	**35.04**	-			-	-		-	
86	+	**34.67**	-		**PP507107**	-	-		-	
87	+	**35.07**	+	**37.68**		-	-		-	
88	+	**35.92**	-		**PP256202**	-	-		-	
89	-		+	**38.35**	**PP101761**					
90	+	**38.14**	-		**PP101771**	-	-		-	
91	-		+	**40.18**	**PP101762**	-	-		-	

ID	qPCR-1*	Ct qPCR-1	qPCR-2*	Ct qPCR-2	GenBank Accesion Number	Faeces global qPCR	qPCR-1	Ct qPCR-1	qPCR-2	Ct qPCR-2
92	+	34.49	-			-	-		-	
93	+	33.75	+	36.50	PP216708					
94	-		+	38.58	PP256203	-	-		-	
95	+	38.86	-		PP256204	-	-		-	
96	+	30.24	+	34.35	PP101763	+	+	40.14	-	
97	+	34.35	-		PP101764	+	+	36.87	-	
98	+	39.95	-		PP507109	+	-		+	39.46
99	+	35.53	+	36.30	PP507110	-	-		-	
100	+	19.91	+	22.44	PP216710	+	+	23.98	+	26.29
101	+	31.16	+	34.63	PP507112	-	-		-	
102	+	36.67	-		NA[†]	-	-		-	
103	-		+	38.86	NA[†]	-	-		-	
104	+	35.09	+	38.41	PP507113	+	+	26.86	+	30.36
105	+	36.32	+	38.20	PP507114					
106	+	20.10	+	23.08	PP216711	+	+	23.72	+	26.13
107	+	39.99	+	40.21		-	-		-	
108	+	18.50	+	20.06	PP471856	-	-		-	
109	+	30.49	+	36.00	PP471857	-	-		-	
110	+	34.88	+	38.96	PP471858	-	-		-	
111	+	29.18	+	32.66	PP471859	-	-		-	
112	+	37.67	-			-	-		-	
113	+	17.70	+	19.84	PP471860	+	+		+	
114	-		+	39.32	PP471861					
115	-		+	36.47	PP471862	-	-		-	
116	-		+	40.91	PP471863	-	-		-	
117	+	35.60	-		PP256205	-	-		-	
118	+	32.49	+	38.1	PP256206	-	-		-	
119	-		+	39.23	PP471850	-	-		-	
120	-		+	39.39		-	-		-	
121	+	37.39	+	36.77	PP256194	-	-		-	
122	-		+	26.13	PP471852					
123	+	36.13	-		PP256195	-	-		-	

ID	qPCR-1*	Ct qPCR-1	qPCR-2*	Ct qPCR-2	GenBank Accesion Number	Faeces global qPCR	qPCR-1	Ct qPCR-1	qPCR-2	Ct qPCR-2
124	+	**22.05**	+	**29.29**		+	+	32.21	+	37.23
125	+	**36.01**	-			+	+	33.77	-	
***Sixty-nine (55.2%) out of the 125 animals were positive by both qPCRs in liver tissue.** NA†: These samples were confirmed by sequencing but these were not submitted to GenBank due to low quality										

References

1. Sridhar S, Yip CC, Wu S, Cai J, Zhang AJX, Leung KH, Chung TWH, Chan JFW, Chan WM, Teng JLL, Au-Yeung, RKH, Cheng VCC, Chen H, Lau SKP, Woo PCY, Xia NS, Lo CM, Yuen KY. Rat hepatitis E virus as cause of persistent hepatitis after liver transplant. Emerg Infec Dis 2018;24(12):2241. https://doi.org/10.3201/eid2412.180937.
2. Suparyatmo JB, Andayani IGAS, Takahashi M, Ohnishi H, Jirintai S, Nagashima S, Nishizaaw T, Okamoto H. Marked genomic heterogeneity of rat hepatitis E virus strains in Indonesia demonstrated on a full-length genome analysis. Virus Res 2014; 179:102-112. https://doi.org/10.1016/j.virusres.2013.10.029.
3. Mulyanto S, Depamede SN, Sriasih M, Takahashi M, Nagashima S, Jirintai S, Nishizawa T, Okamoto H. Frequent detection and characterization of hepatitis E virus variants in wild rats (Rattus rattus) in Indonesia. Arch Virol 2013;158:87-96. https://doi.org/10.1007/s00705-012-1462-0.

4. Rios-Muñoz L, Gonzálvez M, Caballero-Gomez J, Castro-Scholten S, Casares-Jimenez M, Agulló-Ros I, Corona-Mata D, García-Bocanegra I, López-López P, Fajardo T, Mesquita JR, Risalde MA, Rivero-Juárez A, Rivero, A. Detection of Rat Hepatitis E Virus in Pigs, Spain, 2023. Emerg Infec Dis 2024; 30(4):823. https://doi.org/10.3201/eid3004.231629
